# Finite element modelling of thermal and moisture mapping of layered cricket helmets

**DOI:** 10.1016/j.heliyon.2023.e13179

**Published:** 2023-01-25

**Authors:** Z.W. Guan, A.R. Dullah, X.L. Wang, Q.Y. Wang

**Affiliations:** a Advanced Materials Research Centre, Technology Innovation Institute, Abu Dhabi, United Arab Emirates; b School of Mechanical Engineering, Chengdu University, Shiling Town, Chengdu 610106, China; c School of Engineering, University of Liverpool, Liverpool L69 3GQ, UK; d Faculty of Mechanical Engineering, Universiti Teknikal Malaysia Melaka, Malaysia

**Keywords:** Layered cricket helmet, Thermal and moisture mapping, Microclimate, Thermal comfort, Heat transfer, Mass diffusion, Finite element, Microsensor

## Abstract

This paper presents the development of numerical modelling to simulate thermal and moisture mapping of layered cricket helmets. The 3D laser scanning methodology was used to obtain geometrical data of a dummy human head with non-ventilated (NVL) and ventilated (VL) helmets to generate the meshes. Here, heat transfer and mass diffusion were applied in the finite element simulations to model the temperature and relative humidity (RH) distributions inside NVL and VL helmets, which were processed as the temperature-time and RH-time charts. The simulated results were validated against the corresponding experimental measurements with reasonably good correlation, in terms of the general trend on reginal temperature and RH against time, although parameters such as helmet movement and local sweating were not considered in the modelling to simplify the simulation. The discrepancies between the FE simulation results and the measurements are generally within 7% for in-helmet temperature and 5% for RH, for both types of helmets in the low ambient conditions (20 °C and 50% RH), although such the discrepancy is about 10% for the VL helmet subjected to the high ambient conditions (35 °C and 30% RH). The models developed are ready to be used for parametric studies on non-ventilated helmet to optimize the ventilation openings for improving the thermal comfort.

## 1 Introduction

A helmet designed as an item of personal protective equipment (PPE) is vital in practice to protect human head from any occupational or sports hazards. When people use a helmet strength and thermal comfort are two main factors considered. Many types of helmets exist in the current market, which are primarily designed to accommodate impact loading. However, the competitiveness of the helmet most relies on thermal comfort nowadays due to strong materials like composites being applied in the production [1]. Therefore, thermal comfort of a helmet is considered more frequently when a customer purchases a helmet for protection purpose, especially for firemen and strenuous sport players.

Thermal comfort of human head is related to heat and moisture transfer. Lack of ventilation in helmets likely results in heat-stress on the head [[2], [3], [4]]. The use of PPEs like helmets limits the exchange air and evaporation of sweat [5] and creates a microenvironment between the head and the inner surface of a helmet. The heat and moisture trapped in the microclimate causes discomfort to the user [6]. The thermal discomfort increases by increasing the temperature and relative humidity (RH) and in the worst-case scenario likely increases the risk of heat related stress and hyperthermia on the user [5]. As a result, it is necessary to reduce the risk of heat related stress and hyperthermia when a helmet is used in tropical countries where the temperature and RH are high, and in occupations that involve hard physical work in which use of a helmet is required by law (e.g. forestry, fire service, construction) [7,8]. Batting in cricket in hot weather with a cricket helmet on is one of these demanding activities. In sports like cricket where correct and rapid decision making and a high level of attention are essential for batsmen, any thermal discomfort will result in less efficient performance by player [9].

In addition, thermal comfort offered by helmets may influence the willingness of wearer according to the research on effects of ventilated safety helmets [10,11] on the performance. However, there are regulations to enforce users to put on helmets due to their occupations. The accidents may happen if they do not wear a safety helmet due to the discomfort of a helmet. A comparative experimental study of the thermal properties of a cricket helmet was conducted and suggested that helmets impeded heat dissipation form head and caused headform surface temperature to rise by 1.5 ± 0.1 °C around the frontal and parietal regions, which might cause local discomfort [12]. Meanwhile, the type and design of padding may also influence the rate of evaporative heat dissipation from head and face. Hence, the type of material and thickness of the padding is critical for the effectiveness of evaporation heat loss and comfort of wearer [13]. Therefore, thermal comfort of helmets should be considered as a significant factor in design.

The local microclimatic conditions are the most important factors in affecting the thermal sensations and comfort assessments of people [14]. There was a case study using a Computational Fluid Dynamics (CFD) tool to solve the Simplified Thermoregulation Bio-heat (STB) equation to gain a better insight into a comprehensive and transient thermal comfort evaluation [15]. The same tool of CFD was used to deal with convection and diffusion phenomena for water vapour, required for comprehensive thermal comfort analysis and condensation mechanisms [16]. Another study carried out by Fu and his colleagues [17] was to develop a detailed model of heat and moisture transfer through clothing, which was implemented into the multi-segment UC Berkeley Thermophysiological Comfort model (BTCM). This model could be used to predict thermal physiology and thermal comfort with different clothing types in different thermal environments. Therefore, the numerical modelling of thermal and moisture mapping inside cricket helmets is likely an effective tool to be used to simulate the microclimate conditions in close contact with human body and further to assist optimizing the related thermal comfort.

However, the previous studies on the thermal comfort of helmets were primarily restricted to the experimental measurements at few locations due to the unavailability of micro sensors to acquire both temperature and moisture distributions inside a helmet. As the result, computer models developed to simulate the in-helmet microclimate conditions could not be validated properly. Therefore, the work on numerical simulations of thermal and moisture mapping inside a helmet is limited up to date.

The real-time experimental measurements of the in-helmet thermal and moisture mapping on human subjects [18] make the proper validation of numerical simulations to be possible. This paper presents the development of 3D numerical modelling of temperature and relatively humidity distributions inside two types of layered cricket helmets (NVL and VL) in two ambient conditions to fill the current knowledge gap. Here, the temperature distribution is modelled through heat transfer and RH distribution through mass diffusion. The computer simulations are compared with the measured regional temperatures and relative humidity in a chart format, with reasonably good correlation. The validated models are also used to predict the in-helmet temperature and RH distributions. The numerical models developed are ready to be used for further studies on ventilation opening to optimize the thermal comfort not only for layered cricket helmet but other types of head gears.

## 2 Finite element modelling of thermal and moisture mapping

### 2.1 Development of the geometric models of the head-helmet system

**Fig. 1:**
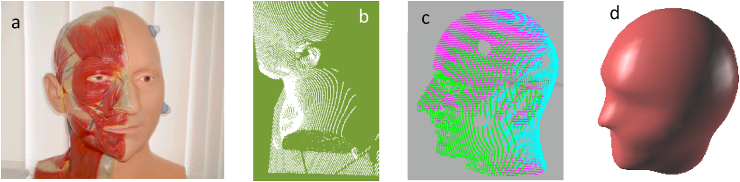
The creation of 3D human head model. (a) head model, (b) the point cloud data, (c) the aligned point cloud assemblies, (d) the final geometric model.

In this study, a portable 3D laser scanner Optix 400 L model (3D Digital Corp. Connecticut USA) based on laser triangulation was used to acquire outer surface data for a dummy human head and a helmet shell. The scanner was connected to a personal computer and Realscan software from 3D Digital Corp was used to acquire point cloud data which were then exported to RHinoceros reverse engineering commercial software (Robert McNeel Seattle USA and Associates) for surface reconstruction. Fig. 1a-d shows the creation of a 3D human head model from the dummy head, the point cloud data, the aligned point cloud assembly to the final geometric model. Scanning was also carried out on two differently ventilated helmets, the Elite (NVL) and the Pro Performance (VL) layered cricket helmets (senior type size 57–62) provided by Gray Nicolls.

To have meshes with a quadrilateral shape for a better computational quality, the helmet model was simplified in certain areas. This includes the omission of the fabric liners, face grill, back head adjuster and chin strap. Then, the 3D human head model created from the scanning process was modified following average data derived from measurements of human subjects recruited for the experimental tests.

### 2.2 Development of computer modelling of the in-helmet microclimate

**Fig. 2:**
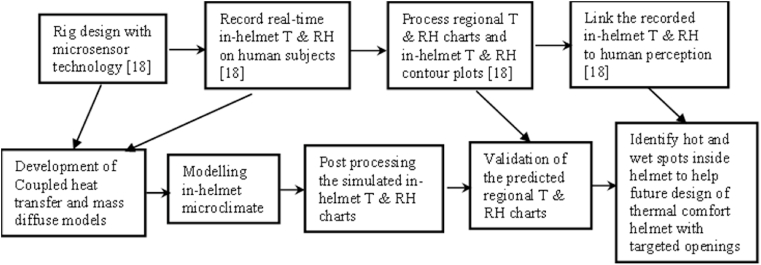
A flow chart of thermal comfort research on cricket helmets.

A flow chart is shown in Fig. 2 to give the development of the computer models to simulate the in-helmet temperature and relative humidity distributions, which is related to the referenced experimental work.

#### 2.2.1 Mesh generation

In this work, a mesh sensitivity study was carried out to select the optimum mesh size for the simulation. The forced convection/diffusion eight-node hexahedral (brick) element (DCC3D8D) available in commercial finite element (FE) code Abaqus [19] with dispersion control was selected instead of the tetrahedron element, as it would provide better stability during numerical analysis. The elements were tested on the simulation of heat transfer only. No mesh sensitivity testing for mass diffusion was carried out as mass diffusion is temperature dependent. The sensitivity studies [1] for the different element sizes were based on a one step simulation (600 s) using the same parameters as those used in the practical simulations, except for that the fixed increment time was set at 100 s and the simulation was set to be stopped when changes from different simulations were less than 0.005 °C.

According to the mesh sensitivity study, the smaller size, i.e. 5 mm or less, was selected for the typical element size used in the critical regions for modelling of both types of helmets, i.e. NVL and VL ones. Likewise, the optimum element size for mass diffusion simulation was also considered to be 5 mm or less. The selected element size was further refined in the regions around the ventilation openings and the peripheral air channels.

**Fig. 3:**
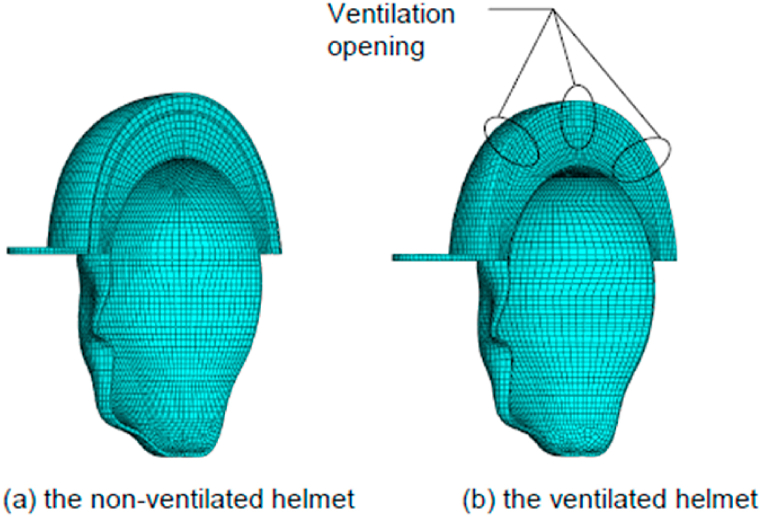
the finite element models of the helmet-head systems.

**Fig. 4:**
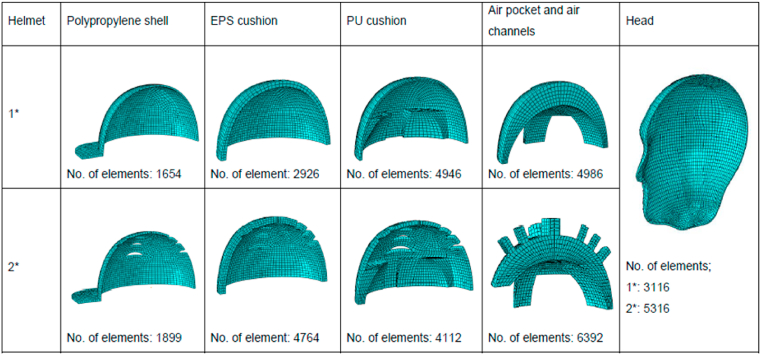
The mesh components of the non-ventilated^1^* and the ventilated^2^* models.

In heat transfer model, the thickness of the PU cushion and EPS foam was assumed to be uniform to simplify the model. For the mass transfer model, the simulation involves the scalp and in-helmet air pocket only, as the other parts are assumed to be non-permeable. The finite element models of both helmets are defined as in Fig. 3a and b and the details are given in Fig. 4.

#### 2.2.2 Heat transfer model

The essential material properties for the simulation of transient heat transfer are density *ρ*, specific heat, *c* and thermal conductivity, *k*, which are assigned to the related geometry entities. The material properties used in the modelling were obtained from existing literature, as listed in Table 1.

**Table 1:** Thermal properties of materials used in the current models.

Part	Density, ρ (kg/m^3^)	Thermal conductivity, k (W/mK)	Specific heat, c (J/kg°C)	Source
Air 20 °C	1.204	0.02514	1007	Cengel 2006 [20]
35 °C	1.145	0.02625	1007	
EPS foam	24	0.023	1600	Cengel 2006 [20]
Polypropylene	900	0.12	1930	Sarda et al. [21]
PU cushion	^a,b^30	^a,b^0.024	^b^1500	a- [20] b- [22]
Scalp	1130	0.384	3570	Janssen et al. 2005 [23]

The initial conditions describe the values of the thermal state of the parts at the beginning of the modelling which are same as those at the beginning of the test. The initial temperatures of all components, except for the scalp, were considered the same as the ambient temperature since the helmet was conditioned at ambient temperature prior to the test. In the practical situation, the initial in-helmet temperature was slightly higher than the ambient temperature even after 30 min conditioning. The scalp temperatures (*t*_*b*_) used are 32 and 36 °C respectively corresponding to the ambient temperatures of 20 and 35 °C [24], with core body temperature being taken as 38 °C.

The boundary conditions are important for the continuity of temperature and heat flux across the material surface. In this composite helmet model, the boundary conditions applied include surface heat flux, surface convection heat flux from the head, radiation from the head and other layers of the helmet, which are in contact with the air, to the ambient, and the internal scalp core temperature.

The surface heat flux was applied to the outer surface of the head towards the air pocket surfaces that were in contact with the head. The heat flux values used were based on the metabolism rate of an average man as obtained from literature. As the head produces more heat than the other parts of the body, the total equivalent heat produced by the head was considered as 15% [25] of the overall body heat production. Table 2 shows the heat flux values used in computer simulations for the standing and walking phases.

**Table 2:** The heat flux values used in simulations.

Condition	Standing	Walking at 1.4 m/s	Source
Equivalent heat production M_t_ (W)	^b^125	^b^265	a- [25] b- [24] c- [26]
^a^Equivalent head heat production (M_t_X15%) M_h_ (W)	18.75	39.75	
Head metabolic rate (M_h_/^a^0.117m^2^) (W/m^2^)	160	340	
Metabolic rate increase due to wearing helmet (W/m^2^)	^c^5	^c^10	
Final heat flux value (simulation) (W/m^2^)	165	350	

Temperature distributions and heat flow between constituent materials are dependent on interaction properties used. The interactions in the FE models involve the scalp, helmet and the in-helmet air, scalp and the environment air. The helmet components interact with each other to make the situation more complex. The effective gap thickness and gap conductance for the interfaces used in the simulations are shown in Table 3.

**Table 3:** The effective gap thickness and gap conductance for the interfaces.

Interface	Gap range (mm)	Effective gap (mm)	Gap conductance (W/m^2^K)	
			20 °C	35 °C
Head-Air	(0–50)	25	1	1.0
Head-PU	(0–5)	2.5	9	9.2
Air-PU	(0–3)	1.5	12	14.3
PU-EPS	(0–25)	12.5	0.5	0.5
EPS-PP	(0–2)	1	18	19.6
Air-Others	–	–	12	14.3

The free and forced convection coefficient values vary between regional head areas. The final free and forced convection coefficients used in the simulation are shown in Table 4.

**Table 4:** Free and forced convection coefficient values used in simulations corresponding to ambient temperature of 20 °C and 35 °C.

Simplified microclimate regions	Natural convection (W/m^2 o^C) Standing at room air movement less than 0.2 m/s $H_{cn}=2.38{\left(t_{b}-t_{a}\right)}^{0.25}$		Walking on treadmill at a speed of 1.39 m/s, $H_{cf}=8.6\sqrt{v}$
	t_a_ = 20 °C H_cn_ = 4.43	t_a_ = 35 °C H_cn_ = 2.38	H_cf_ = 10.14
Frontal	*0.8H*_*cn*_ = 3.52	*0.8H*_*cn*_ = 1.44	*0.15H*_*cf*_ = 1.53
Lateral	*0.4H*_*cn*_ = 1.76	*0.4H*_*cn*_ = 0.96	*0.20H*_*cf*_ = 2.04
Top	*0.5H*_*cn*_ = 2.20	*0.5H*_*cn*_ = 0.72	*0.15H*_*cf*_ = 1.53
Rear	*1.6H*_*cn*_ = 7.04	*1.6H*_*cn*_ = 3.60	*0.60H*_*cf*_ = 6.12
Outer	*H*_*cf*_ = 10.14	*H*_*cf*_ = 10.14	*H*_*cf*_ = 10.14

The radiation in the simulation is realized by the surface radiation to the ambient. The radiation emitted by a body and helmet surface to the surrounding is represented by the emissivity of a surface, ε, which is the ratio of the radiation emitted by a surface at a given temperature to the radiation emitted by a black body at the same temperature. The radiation takes place at the interfaces between different parts. Table 5 shows the emissivity values used in the simulation.

**Table 5:** Emissivity values of different materials used in simulations.

Part	Equivalent assumption	Emissivity, ε at 300 K	Reference
Expanded polystyrene	Cloth	0.87	Cengel 2006 [20]
Polypropylene (Elite helmet)	Cloth	0.87	
Polypropylene (PP helmet)	Oil Paint	0.94	
Polyurethane foam	Cloth	0.87	
Scalp	Human skin	0.95	

Simulations were run in six transient steps which represented three alternate standing and walking sessions. Each step was 600 s (10 min) long. Equation (1) was used to calculate the time increment of different model constituents for an element length of 0.005 m to decide a suitable value to be used in the simulation. The calculation results of different materials with three element lengths are shown in Table 6. Based on the table, the minimum time increment to be used to avoid spurious oscillations is 60.3 s for polypropylene. However, since the element length in certain areas was more than 0.005 m, the time increment was then set at 100 s.

(1) $$\Delta t>\frac{\rho c}{6k}\Delta l^{2}$$

where Δt is time increment and *l* is an average element length.

**Table 6:** Minimum time increments for different materials.

Element length (mm)	Δt (s)		
	5	6	7
Air 20 °C	0.20	0.29	0.41
Air 35 °C	0.18	0.26	0.36
Expanded polystyrene	7.00	10.00	13.60
Polypropylene	60.30	86.50	118.20
Polyurethane foam	7.00	11.20	15.30
Scalp	45.70	63.00	85.80

#### 2.2.3 Mass diffusion model

The diffusion of water vapour from the head surface to the surrounding environment is a complicated process as it involves interaction of many parameters like air and body temperatures, RH, air flow and the helmet internal lining materials. In order to simplify the mass diffusion analysis, only air and scalp were modelled and other parts were considered to be non-permeable. Also, air flow was not considered in the model.

Here, the normalized concentration term of parts per million (ppm) was used in the mass diffusion simulation. The mass concentration to RH conversion is shown in Eq. (2).

(2) $$C_{H_{2}O}=X_{H_{2}O}∙n_{air}∙M_{W}$$

where,

$$C_{H_{2}O}-mass\;concentration\;of\;water\;\left(g/m^{3}\right)\text{,}$$

$$X_{H_{2}O}-mole\;fraction\;\left(volume\;mixing\;ratio\;of\;water\right)\;or\;RH\text{,}$$

$$n_{air}-air\;density\;\left(mol/m^{3}\right)\text{,}$$

$$M_{w}-molecular\;weight\;of\;water\;vapour\;\left(18.02\;g/mol\right)\text{.}$$

In this case, $X_{H_{2}O}$ is considered as RH.

To carry out numerical modelling of the transfer of sweat vapour inside the helmet, the relevant material properties, initial conditions, interaction between materials, and boundary conditions need to be determined.

Apart from the heat diffusive properties like density, specific heat and conductivity, mass diffusivity and solubility need to be defined in the numerical modelling of mass diffusion, as they control the diffusion or movement of one material through another. Solubility represents the maximum amount of material that can be dissolved in a liquid at a specific temperature [20] and measured in g/g basis or kg/kg basis. The solubility, *s*, of a material during the diffusing phase in a mass diffusion process is defined in commercial code ABAQUS [19] in Eq. (3) as follows.

(3) $$s=\frac{\emptyset }{C}$$

where ∅ is a normalized concentration and *C* is concentration. The values of diffusivity and the solubility values are shown in Table 7.

**Table 7:** Diffusivity and solubility values used during simulation.

Part	Diffusivity (m^2^/s)	Solubility	Source
Air 20 °C	^a^2.50 × 10^−5^	5.10 × 10^2^	a – [20]
Air 35 °C	^a^2.68 × 10^−5^	2.67 × 10^2^	
Scalp	^b^1.60 × 10^−7^	2.67 × 10^2^	b – [27]

The temperature values used in the mass diffusion model were read from the output file corresponding to the step of the heat transfer analyses as the nodes in the mass diffusion and heat transfer analysis were the same.

The only interaction during mass diffusion is the conduction and diffusion between the head surface and the surface of the internal air pocket. As mass diffusion is considered to occur only when there is a mass concentration gradient, the boundary conditions need to be applied at the head surfaces to limit the concentration distribution during every step. The boundary conditions at the head surface inside the helmet were set at 58160 ppm, whilst for the head surface outside the helmet the value varied between steps. The values were based on the average RH readings taken at the end of each step from the experimental tests on the non-ventilated helmet subjected to moderate and high ambient conditions. The RHs were converted to normalized concentration in two steps. The first step involved the conversion of RH to mass concentration (g/m^3^) by using the online air humidity converter software [28]. The temperatures used for this conversion were taken from the average temperatures through the corresponding heat transfer analysis. The mass concentration was then converted into ppm unit by using the online ppm converter software [29]. The boundary conditions for the air gaps and air channels interfacing with the outside air were set to be the ambient RH. The boundary conditions used at every step during the moderate and high ambient simulations of both NVL helmet and VL helmet models are shown in Table 8.

**Table 8:** RH and normalized concentration conversion for mass diffusion simulations.

Step	Relative humidity (% RH)	Normalized concentration (ppm)	
		20 °C/50% RH	35 °C/30% RH
Initial condition	50/30	10980	14950
1	70	21120	36460
2	75	23660	38760
3	80	26322	42120
4	85	27720	44020
5	87.5	29340	46160
6	90	29680	46630

The mass fluxes applied on the surface of the head were based on the sweat production rate taken from De Bruyne et al. [30]. The sweat production rates at low effort and high effort were used for the standing and walking phases, respectively. The tests conducted were processed at the frontal, lateral, rear centre and rear lateral regions. According to the test results, the highest sweat production rate was shown at the rear centre. However, the average of the rear centre and rear lateral were used for the rear region in the simulation. The frontal and lateral sweat production rates were similar. The sweat rate on the top of the head was considered the same as the frontal and lateral regions due to the similarity of the temperature in the frontal, lateral and top regions. On top of that, the face and back of the neck were also considered to share the same sweat production rate with the frontal, lateral and top regions.

Table 9 shows the mass loads applied to different parts of the head during standing and walking sessions. The same loads were used for the simulations of the non-ventilated and ventilated helmets at 20 °C and 35 °C.

**Table 9:** Mass flux values at different head surface areas used during the simulation.

Area	Mass fluxes applied (mg/min.cm^2^)	
	Standing	Walking
Frontal	0.55	1.2
Lateral	0.55	1.2
Top	0.55	1.2
Rear	0.75	1.7
Face/back of neck	0.55	1.2

Similar to the heat transfer simulation, the mass diffusion simulations were also run in six steps which represent three alternate standing and walking sessions. A similar time increment, i.e. 100 s, was also used.

When collect in-helmet temperature and relative humidity data to validate computer models, fifteen healthy student subjects with similar hair styles and amounts of hair were recruited for testing the NVL and the VL helmets in two ambient conditions, i.e. the moderate and the high one. Their head size (length, breadth and circumference) was measured beforehand to ensure that the helmet would fit. Related information like height, weight and age of the selected subjects was also recorded into the subjective perception form [1]. The subjects were released by the University of Liverpool Ethics committee for the test and signed a consent form.

## 3 Modelling results and discussion

### 3.1 Simulation of the in-helmet temperature and RH at moderate ambient conditions (20 °C and 50% RH)

**Fig. 5:**
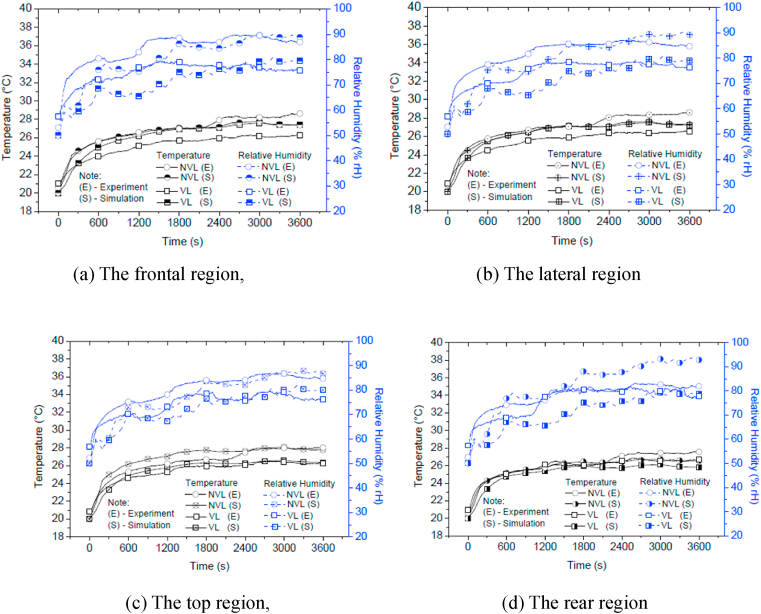
Comparison of the experimentally measured and numerically simulated temperature and RH of the NVL and the VL helmets in the ambient condition of 20 °C and 50% RH.

Fig. 5(a), (b), (c) and (d) show the comparisons of the experimentally measured and numerically simulated temperature and RH in the four simplified regions (frontal, lateral, top and rear regions) for the NVL and the VL helmets. The overall features of the experimental results were simulated well. The experimental measurements show that the in-helmet temperatures for the VL helmet are generally lower than those for the NVL helmet, which was well simulated. The simulations show that the temperatures in all regions initially increase significantly once a helmet is put on until they reach an initial equilibrium at t = 600 s, due to the higher temperature of the head surface. After that, the overall increasing rate of the temperature decreases significantly as the temperature gradient between the head surface and the interior surface of helmet where sensors are located becomes lower with increasing time. The temperature may be divided into three varying stages, i.e. (1) the initial phase of high increase (0–600 s), (2) the second phase of mild increase (600–1800 s), and (3) the third phase of small increase (1800–3600 s). Such performance was determined by the mild ambient temperature and the mild activity.

Since the head skin temperature is much higher than the ambient one, the microclimate temperature inside the helmet keeps increasing until it reaches a final equilibrium condition. After that, the regional temperatures fluctuate around the balanced temperature, which may be taken as the average value of all in-helmet temperature readings. From Fig. 5, temperature readings by the time of 3600 s may have entered the final equilibrium stage. In general, correlation between the measured and predicted in-helmet temperature is good, with an error within 7% throughout the measuring time period for all regions.

The simulated regional RHs for both the helmets (Fig. 5) also increase significantly in the first 100 s but drop about 5% before they increase again. The predicted RHs are the conversion results of the normalized concentration that depends on the predicted temperature values. Since the significant increase in the normalized concentration values takes about 600 s but the significant increase on the temperature only takes about 300 s, the lower RH is then calculated.

Fig. 5 indicates that the predicted RHs increase in a similar fashion of the experimental one in all simplified regions. Such the trend remains until the end of the testing. In general, the predicted RHs correlate to the measured ones reasonably well for both helmets. The largest discrepancy is about 10% at the rear region for the NVL helmet model at t = 3600 s and the VL helmet model at t = 1200 s. The likely reason is that the air exchange (so the water vapour transfer) between the in-helmet air pocket and the ambient environment was not simulated efficiently since the factors such as air movement and moisture absorption by the internal fabric material were not considered.

In general, correlation between the simulated and experimental RHs is reasonable in terms of both the value and the trend, with majority of discrepancies within 5%. The simulated RHs for the VL helmet are always lower than those for the NVL helmet, which is also true for the corresponding test results. Almost all RH predictions at all microclimate regions for the NVL helmet are within the experimental result boundary. However, the simulated RHs in the VL helmet are outside the experimental result boundary for most of the period considered, nevertheless such a gap is reduced towards the end. This is partially due to the 1 °C lower initial temperature in the modelling (based on the ambient temperature) in comparison to the corresponding test results since the normalized concentration (so RH) is dependent upon temperature.

### 3.2 Simulation of the in-helmet temperature and RH at high ambient conditions (35 °C and 30% RH)

**Fig. 6:**
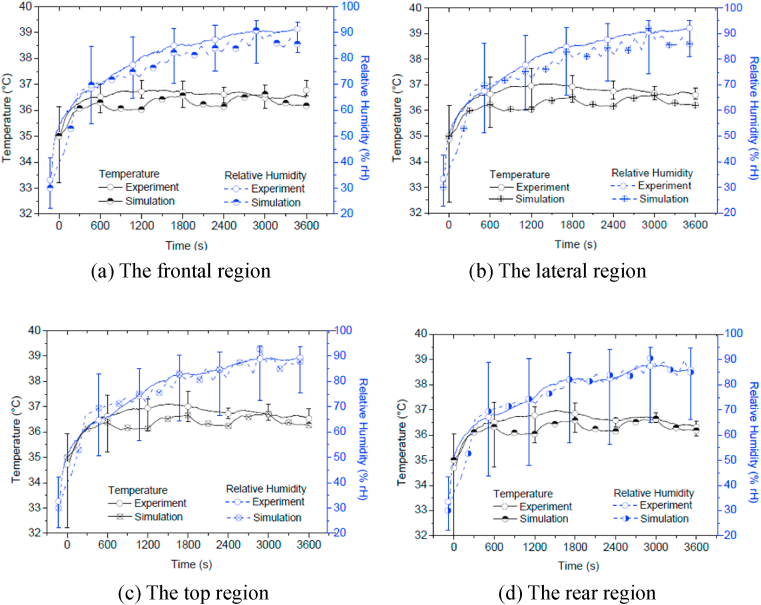
Simulated and measured temperature and relative humidity in the simplified regions for the NVL helmet in the ambient condition of 35 °C and 30% RH.

The simulated temperatures and RHs of the NVL helmet are compared with the experimental results in the same chart for better comparison. Fig. 6(a), (b), (c) and (d) show the comparisons of temperatures and RHs between the simulated and experimental results with standard deviation at the four simplified regions. The figure shows that the initial temperature simulated is almost similar to the experimental one, whilst the RH shows 5% difference. The difference between the ambient temperature of 35 °C and the head skin temperature of 36 °C is only 1 °C. Although the core body temperature is set to 38 °C, the increase in the in-helmet temperature is limited. This is reflected by both the experimental measurements and the numerical simulations, which show good correlation as indicated in almost all regions.

The simulated temperatures in all regions are lower than the experimental ones. During the walking and resting phases, the measured temperatures drop and then increase slightly respectively, which is captured by the numerical model. The measured regional temperatures at the frontal and the top areas show a slight decrease after reaching an initial equilibrium due to relatively high sweating effects there. However, such a decrease was not modelled in the simulation, as the heat transfer model did not include water vapour from sweating. The equilibrium temperature is a dynamic one and varies from time to time.

In general, the simulated temperatures are lower than the experimental ones, due to the fact of that no human thermoregulation was simulated, but the differences between them are reduced towards the end of the test (also simulation) duration. The temperatures at all regions during the standing sessions are within the lower bound of the standard deviation; however, for the walking sessions, the temperatures are beyond the limit. This is likely attributed to the lack of pumping effect from air movement, since such the movement was not simulated. The highest discrepancy is shown in the lateral region at t = 900 s with a value of about 2 °C, i.e. 6 %.

The RHs at all regions are well predicted as they are within the standard deviation limits. The RHs predicted at the frontal, lateral and top regions are slightly lower than the experimental ones. However, the RH predictions at the rear region almost match the experiment RHs there. The highest discrepancy is shown at the frontal region at t = 300 s with the value of 10% RH. The good RH predictions are helped by the lack of ventilation openings on the NVL helmet, which gives limited air movement inside the helmet.

According to Fig. 6, the temperatures at the microclimate regions vary by between 0.1 and 0.2 °C and there are almost no differences in the RH values predicted due to the very limited ventilation in the NVL helmet.

**Fig. 7:**
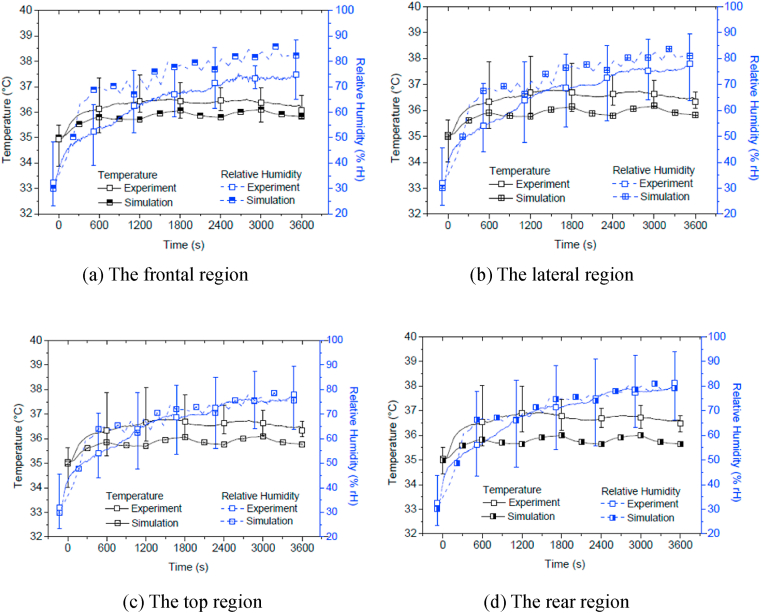
The simulated and measured temperature and relative humidity in the simplified regions for the VL helmet subjected to the ambient condition of 35 °C and 30%RH.

Fig. 7(a), (b), (c) and (d) show the comparisons between the simulated and measured results at the four simplified regions for the VL helmet. The figures indicate that the initial temperatures simulated for all regions correlate well with the experimental ones. The general patterns of the temperature curve plots predicted for all regions are similar to the experiment results, although the simulations are lower than the experiment ones in overall. The simulated temperatures are generally within the low deviation limit during the standing session but outside the limit during the walking session, except for the rear region. The smallest temperature gap of 0.5 °C is at the frontal region (Fig. 6a) at t = 3000 s, whilst the largest temperature gap of 1.5 °C is at the rear region (Fig. 7d). Clearly, the variation in the simulated in-helmet temperatures and RHs from region to region is small, due to the low temperature gradient between the in-helmet microclimate and the ambient environment which are linked by ample ventilation openings.

The RH predictions are in good agreement with the experimental results except for those at the frontal region (Fig. 7a) and part of the lateral region (Fig. 7b). Moisture movement and sweat absorption at those regions are quite complicated but were only simulated approximately. The highest discrepancy is shown at the time interval of 600 s at the frontal region with a value of nearly 20% RH. However, such discrepancies are reduced towards the end of the test/simulation, with values less than 5%, except for that at the front region. Good predictions are shown specifically at the top and the rear regions (Fig. 7c and d).

The reasonable predictions of the microclimate temperature and RH for both the non-ventilated and the ventilated helmets subjected to different ambient conditions demonstrate the potential of the numerical models developed. Comparing the simulations from the VL helmet model with those from the NVL helmet model, the former gives a better correlation to the corresponding experimental results. The latter model also reproduces the high temperatures inside the air pocket due to heat accumulation as there are limited heat escape routes between the head and the lower circumferential boundary of the NVL helmet. Such a situation contributes to the relatively hot top regional area (Fig. 6c) in comparison to other regional areas in the NVL helmet model. The predictions of the temperature and RH in the top region (Fig. 7c) for the VL helmet, together with the corresponding measurements there, show relatively low readings in comparison to the surrounding areas. From numerical modelling, this again indicates necessary ventilation openings there to allow the exchange of heat with the ambient atmosphere and to avoid accumulation of heat there. Also, both predictions and measurements show relatively high temperatures and RHs at the frontal and lateral regions, which hints at the necessity for proper ventilation openings to be set there.

Generally, the heat release from the head surface and transfer to the in-helmet microclimate is almost realized by convection since conduction and radiation are limited by the low conductivity property of the constituent materials of the helmets studied and the small air channels to the outside of the helmet. The numerical models developed can produce reasonable predictions of the temperature and RH inside a helmet.

**Fig. 8:**
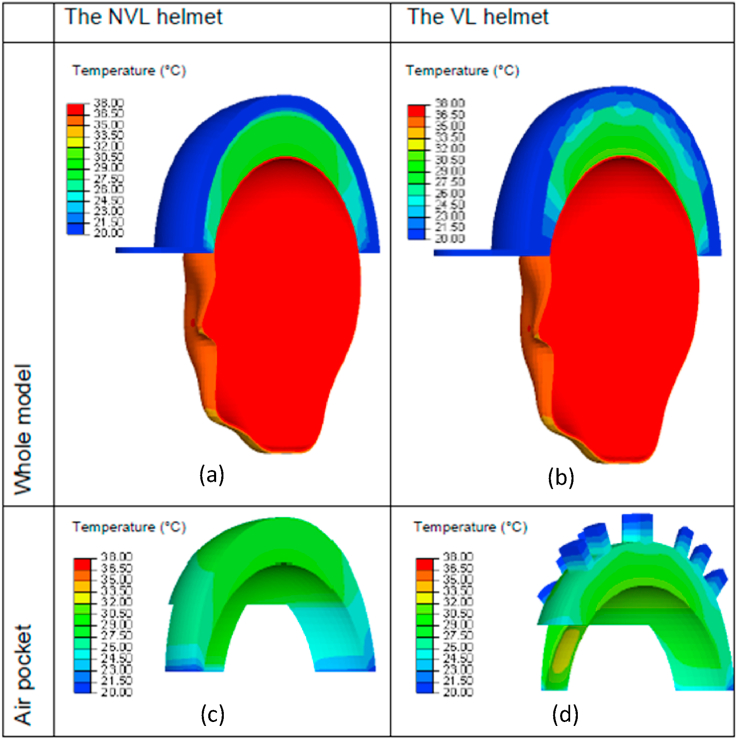
Predicted microclimate temperature distributions of two typical helmets at t = 1800s, subjected to the ambient temperature of 20 °C: (a) the whole model of NVL helmet, (b) the whole model of VL helmet, (c) the air pocket for NVL helmet, (d) the air pocket for VL helmet.

**Fig. 9:**
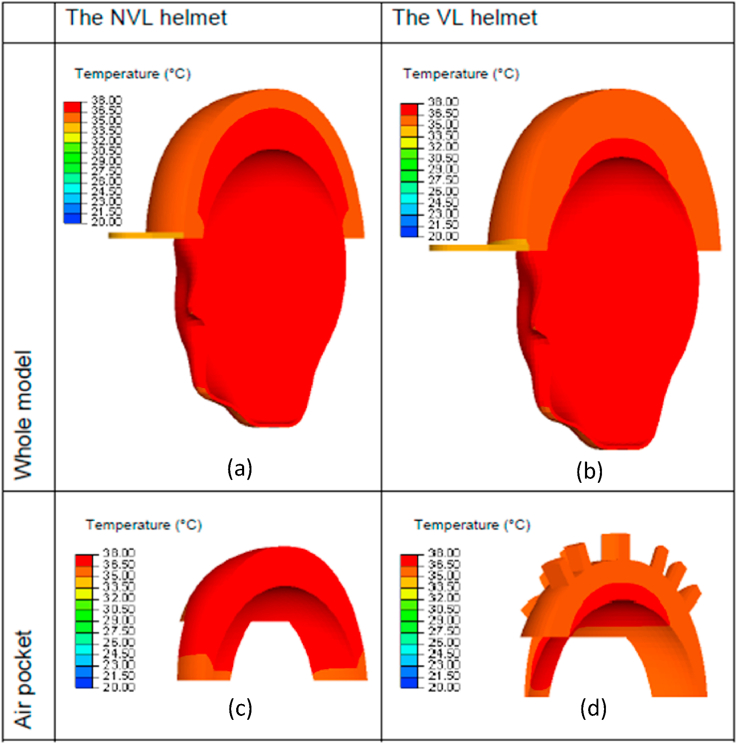
Predicted microclimate temperature distributions of two typical helmets at t = 1800s, subjected to the ambient temperature of 35 °C: (a) the whole model of NVL helmet, (b) the whole model of VL helmet, (c) the air pocket for NVL helmet, (d) the air pocket for VL helmet.

Using the validated models, the temperature distributions of the microclimate environments for the non-ventilated (NVL) and the ventilated (VL) helmets at t = 1800 s (half-way through the measurements) are simulated, which are shown in Figs. 8 and 9, corresponding to the ambient temperatures of 20 °C and 35 °C respectively. The figures show the distributions of temperature inside the air pocket of the NVL and the VL helmets subjected to moderate and high ambient temperature conditions. Here, the hot and cool colour contours of the air pocket are located respectively near the heat source (head) and the microclimate outer surface near the air channels and ventilation openings. The comparisons between the simulations from the two models in both ambient conditions show that the temperature at the outer air pocket surface of the VL helmet is lower than that of the NVL helmet. This is attributed to the availability of ventilation opening air channels through the former helmet that helps to re-distribute the heat to the surrounding environment with a lower temperature. This, in return, reduces the temperature at the outer air pocket surface, especially near the ventilation opening areas.

**Fig. 10:**
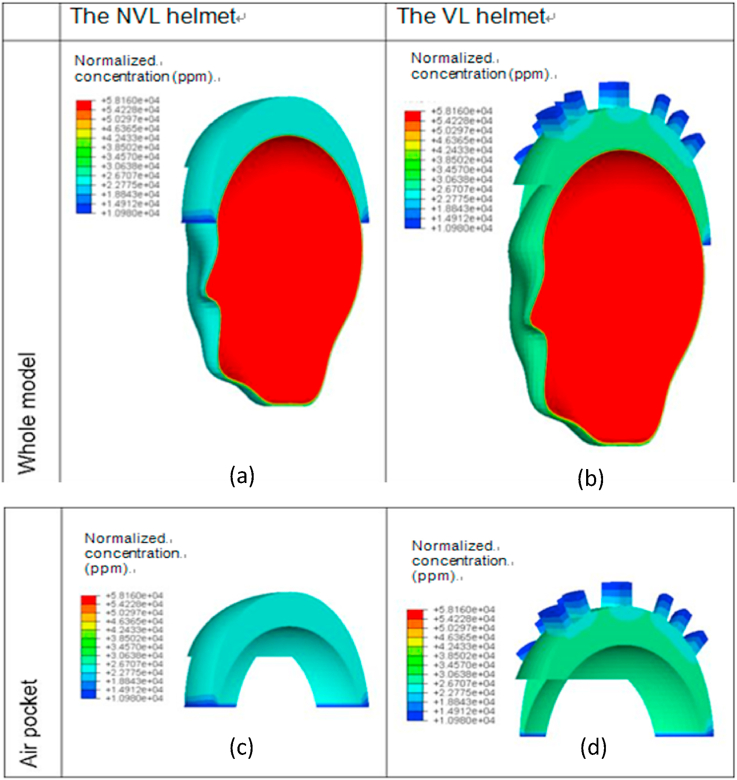
Predicted normalized concentration distributions in the in-helmet microclimate at t = 1800 s for two typical helmets in the ambient condition of 50% RH and 20 °C: (a) the whole model of NVL helmet, (b) the whole model of VL helmet, (c) the air pocket for NVL helmet, (d) the air pocket for VL helmet.

**Fig. 11:**
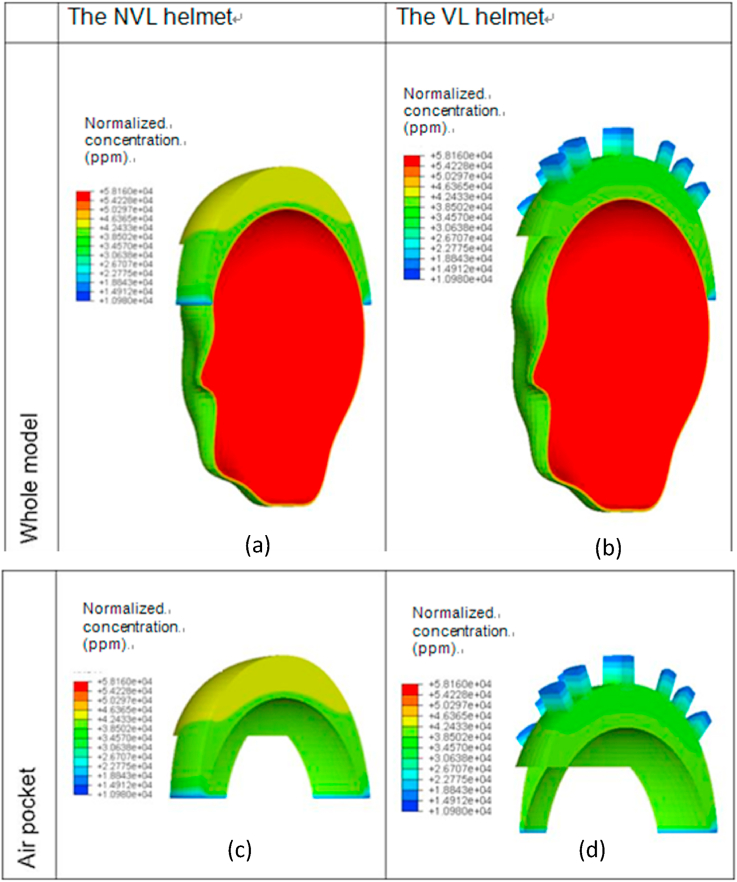
Predicted normalized concentration distributions in the in-helmet microclimate at t = 1800 s for two typical helmets in the ambient condition of 30% RH and 35 °C: (a) the whole model of NVL helmet, (b) the whole model of VL helmet, (c) the air pocket for NVL helmet, (d) the air pocket for VL helmet.

Figs. 10 and 11 show the predicted distributions of the normalized concentration at the time of 1800 s for both the NVL and the VL helmets in the ambient conditions of 50%RH/20 °C and 30%RH/35 °C, respectively. The distribution figures show that the normalized concentration in the regions near to the peripheral and ventilation opening air channels is 50–60% lower than those of the other regions. This is due to the interaction of the air exchange between the air of the microclimate environment inside the helmet and the fresh air outside the helmet, which in return reduces the concentration. The comparisons between the simulations from the two models show that the normalized concentration at the top surface of the air pocket for the NVL helmet is much higher than that of the VL helmet subjected to both the ambient conditions. The reason is again due to the availability of ventilation opening air channels in the latter helmet.

The current modelling on the in-helmet relative humidity relies on the predicted in-helmet temperature distributions as the input data. Ideally, a user-defined subroutine needs to be developed to take temperature-moisture interaction into account more cohesively throughout the iterations. In addition, air movement and moisture absorption by the internal fabric material need to be considered. Furthermore, the predicted in-helmet microclimate conditions are required to be related and correlated to human perception measured experimentally.

## 4 Conclusions

The 3D geometric models of the layered cricket helmets, a human head model, and the air pockets inside the helmet have been successfully constructed using digital laser scanning technology. With the mesh created from the geometric models, heat transfer and the mass diffusion models have been developed for both non-ventilated and ventilated helmets subjected to two different ambient conditions.

The microclimate conditions, in terms of the in-helmet temperature and relative humidity, are successfully predicted and validated against the related experimental measurements. Both temperature and RH variation in the microclimate environment at various time intervals are predicted. Also, variation of the regional temperature and RH for the whole testing period is produced, showing reasonably good agreement with the experimental results, with the error up to 7% for in-helmet temperature and 5% for RH for both helmets in the low ambient conditions (20 °C and 50% RH), although such the discrepancy is about 10% for the VL helmet subjected to the high ambient conditions (35 °C and 30% RH). Using the validated models, the in-helmet temperature and RH distributions are well predicted for both types of helmets subjected to two ambient conditions.

The current models developed may be used to study the microclimate temperature and RH inside a helmet and thus to assist optimizing the thermal comfort of a helmet. However, a user-defined subroutine needs to be developed to take temperature-moisture interaction into account more cohesively throughout the modelling, together with consideration of air movement and moisture absorption by the internal fabric materials.

## Author contribution statement

Z.W. Guan: Conceived and designed the experiments; Wrote the paper.

A.R. Dullah: Performed the experiments; Wrote the paper.

X.L. Wang: Analyzed and interpreted the data; Wrote the paper.

Q.Y. Wang: Contributed reagents, materials, analysis tools or data.

## Funding statement

This research did not receive any specific grant from funding agencies in the public, commercial, or not-for-profit sectors.

## Data availability statement

Data will be made available on request.

## Declaration of interest’s statement

The authors declare no conflict of interest.
